# Systematic review and meta-analysis of the antidepressant-like effects of ginsenosides in animal models: comparative behavioral profiles and exploratory dose-stratified analysis

**DOI:** 10.3389/fphar.2026.1833994

**Published:** 2026-09-17

**Authors:** Yuhua Wu, Yuancheng Jin, Lijiang Wang, Mengfei Dai, Zhujin Song

**Affiliations:** 1 Department of Pharmacy, The Fourth Affiliated Hospital of School of Medicine, and International School of Medicine, International Institutes of Medicine, Zhejiang University, Yiwu, China; 2 Department of Pharmacy, Zhejiang Cancer Hospital, Hangzhou Institute of Medicine (HIM), Chinese Academy of Sciences, Hangzhou, China

**Keywords:** behavior, depression, ginsenosides, mechanism, meta-analysis

## Abstract

**Objectives:**

This study systematically evaluated the antidepressant-like effects of individual ginsenoside monomers and total ginsenosides in animal models of depression, compared pooled behavioral profiles across components, and summarized the principal mechanisms proposed in the included studies. The comparative analyses were intended to identify patterns within the existing evidence base rather than to establish a definitive hierarchy of antidepressant potency.

**Methods:**

Animal studies comparing a ginsenoside intervention with a vehicle-treated depression-model control were synthesized using random-effects meta-analysis. Standardized mean differences (SMDs) were calculated for the sucrose preference test (SPT), forced swim test (FST), tail suspension test (TST), and open field test (OFT) outcomes. Interaction analyses were used to explore component-by-behavior differences, and dose-stratified analyses of ginsenoside Rb1 (GRb1) were considered exploratory.

**Results:**

Twenty-five animal studies evaluating 10 individual ginsenoside monomers and total ginsenosides were included. Ginsenoside treatment increased sucrose consumption in the SPT (SMD: 3.20, 95% CI: 2.61–3.80; Z = 10.52; *p* < 0.00001) and reduced immobility time in the FST (SMD: −2.21, 95% CI: −2.66 to −1.76; Z = 9.72; *p* < 0.00001) and TST (SMD: −2.07, 95% CI: −2.45 to −1.68; Z = 10.44; *p* < 0.00001). GRb1 showed comparatively large pooled effects in the SPT and FST, whereas GRh2 showed strong effects in behavioral-despair paradigms. Other monomers showed notable effects in selected behavioral domains. Interaction analyses suggested that the observed effects varied according to ginsenoside component and behavioral outcome. However, these comparisons were predominantly indirect and were based on heterogeneous experiments. Several pooled SMDs were very large and should be interpreted in the context of small samples, methodological limitations, and possible small-study or reporting effects.

**Conclusion:**

Ginsenosides were associated with improvements across several depression-related behavioral domains in animal models. The evidence supports further investigation of GRb1 and other active monomers in standardized, adequately powered, head-to-head preclinical studies. It does not establish the superiority of one ginsenoside, clinical antidepressant efficacy, clinical safety, or an optimal therapeutic dose.

**Systematic Review Registration:**

https://www.crd.york.ac.uk/prospero/display_record.php?ID=CRD420251273532; Idetifier: CRD420251273532.

## Introduction

Depression is one of the most prevalent and disabling psychiatric disorders worldwide. Its core clinical manifestations include persistent low mood, anhedonia, cognitive impairment, reduced energy, and diminished motivation, often accompanied by irritability, fatigue, and a broad range of somatic symptoms. In addition to markedly impairing quality of life, social functioning, and interpersonal relationships, depression is strongly associated with an increased risk of suicide (Herrman et al., 2019; Uchida et al., 2018). According to the World Health Organization, more than 350 million individuals worldwide are affected by depression, and its prevalence has increased substantially over the past decade. In China, a national mental health survey estimated that more than 95 million individuals are affected, with a lifetime prevalence of 6.8%. Depression disrupts cognitive, emotional, and neural function (Hammen, 2018), frequently co-occurs with sleep disturbance, appetite loss, and other somatic symptoms (Jiang et al., 2024), and is associated with an increased incidence of multiple medical comorbidities (Jiang et al., 2014; Yoshimura and Watanabe, 2025; Zhang et al., 2018). Together, these features make depression a major contributor to the global health burden (Xu et al., 2025; Zhang et al., 2024). Despite extensive investigation, the mechanisms underlying the onset, persistence, and remission of depression remain incompletely understood, reflecting the complex interplay of biological, psychological, and sociocultural determinants.

Current pharmacological treatment for depression relies primarily on tricyclic antidepressants, selective serotonin reuptake inhibitors, and serotonin-norepinephrine reuptake inhibitors. Although these agents provide symptomatic benefit for many patients, their therapeutic use is limited by several important factors. A substantial proportion of patients have an inadequate response or develop treatment resistance, while adverse effects such as nausea, headache, weight gain, sexual dysfunction, and sleep disturbances can impair long-term adherence and treatment outcomes (Kishi et al., 2023; Siwek et al., 2023; Solmi et al., 2020). The global antidepressant market was projected to reach 5.79 billion dollars by 2025 (Kim and Cho, 2021), and antidepressants are also widely used for anxiety disorders, post-traumatic stress disorder, and somatic symptom disorders. These considerations underscore the need to identify new therapeutic candidates with improved tolerability and broader mechanisms of action. Accordingly, natural compounds with favorable safety profiles and potential neuropsychopharmacological activity have received increasing attention in translational research.

Panax ginseng C.A. Meyer has been used for more than 2,000 years in China and other East Asian regions for the management of a wide range of conditions, including diabetes, cardiovascular disease, cognitive impairment, and influenza (Zhou et al., 2023). In Traditional Chinese Medicine, ginseng is regarded as a medicinal herb that can “tonify qi and calm the spirit” and has therefore been used to alleviate symptoms related to depressive disorders (Hou et al., 2020). The principal bioactive constituents of ginseng are ginsenosides, a class of triterpenoid saponins that are commonly categorized into protopanaxadiol (PPD)-type (Ra1-Ra3, Rb1, Rb2, Rc, Rd, Rg3, Rh2, Rh3), protopanaxatriol (PPT)-type (Re, Rf, Rg1, Rg2, Rh1), oleanolic acid-type (Ro), and ocotillol-type compounds (Kim et al., 2017; Yu et al., 2019). Accumulating preclinical studies suggest that ginsenosides may exert antidepressant-like effects through multiple biological pathways. However, the available literature remains highly heterogeneous with respect to animal species, depression models, dosing regimens, administration routes, treatment duration, and behavioral assessment methods. Such variability may contribute to inconsistent effect estimates and divergent mechanistic interpretations. In addition, many studies are limited by relatively small sample sizes, which may reduce statistical power and increase uncertainty regarding the magnitude of the reported effects.

Against this background, the present systematic review and meta-analysis was undertaken to provide a more differentiated synthesis of the preclinical evidence. Previous reviews have established the general antidepressant-like activity of ginsenosides, whereas the current study extends that evidence by comparing multiple monomers and total ginsenosides across distinct behavioral domains, testing whether pooled effects vary by component and outcome, and examining exploratory dose-stratified findings for GRb1. The interaction analyses offer translational value by identifying compounds and behavioral domains that warrant standardized direct comparison; they do not substitute for head-to-head experiments and were not intended to rank antidepressant potency. The review also evaluates the coherence and evidentiary strength of the principal mechanisms proposed in the included studies, with emphasis on neuroplasticity, neuroinflammation, oxidative stress, hypothalamic-pituitary-adrenal (HPA)-axis regulation, and gut-brain signaling.

## Materials and methods

This meta-analysis was prospectively registered in PROSPERO (CRD420251273532). And the meta-analysis was conducted in accordance with the PRISMA guideline, with reporting informed by the PRISMA 2024 Statement (Page et al., 2021).

### Search strategy

Two authors (YH-W and ZJ-S) independently conducted a comprehensive literature search in PubMed, Embase, ScienceDirect, and Web of Science for original research articles published up to 31 October 2025, that investigated ginsenosides in relation to depression. The search strategy combined controlled vocabulary terms (including MeSH terms and Emtree headings) with free-text keywords related to “depression,” “depressive disorder,” “mice,” “ginsenoside,” “ginseng,” and “Renshen.” Full search details are provided in Figure 1. The electronic search was supplemented by manual screening of relevant articles and citation tracking to identify additional eligible studies.

**FIGURE 1:**
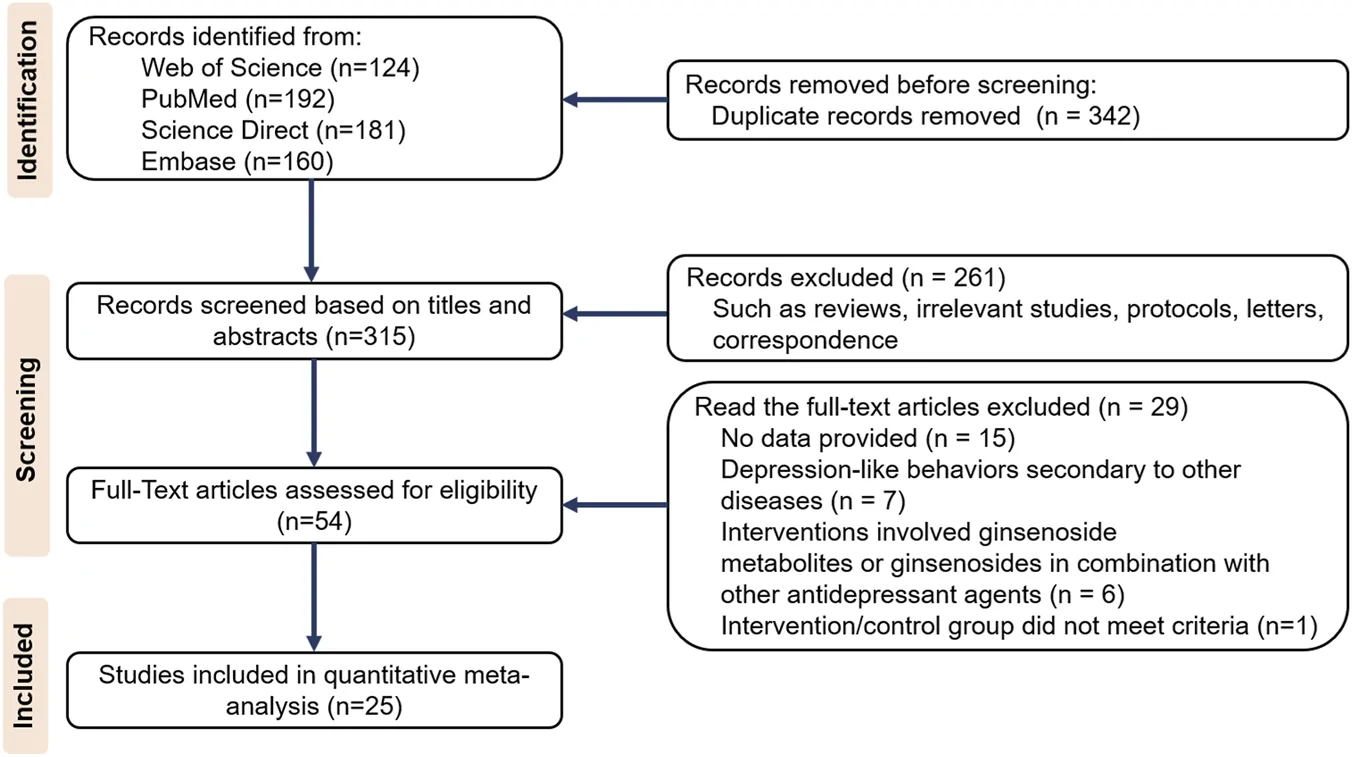
Flow diagram for selection of eligible studies.

### Study selection

Two authors (YC-J and LJ-W) independently screened titles and abstracts and then assessed full-text articles according to predefined inclusion and exclusion criteria, with the aim of evaluating the effects of ginsenosides on behavioral outcomes in animal models of depression. No restrictions were imposed on language, publication year, or study design. Studies were included if they met all of the following criteria: (1) they used an animal model of depression, irrespective of animal age, sex, or modeling duration; (2) the intervention consisted of ginsenoside monomers or total ginsenosides, regardless of route, frequency, or dosage; (3) a vehicle-treated model control group was included; and (4) at least one behavioral test was performed. Studies were excluded if they: (1) were case reports, conference abstracts, commentaries, reviews, or editorials; (2) did not include behavioral testing; (3) evaluated post-digestion metabolites of ginsenosides or combined ginsenosides with other pharmacological agents; or (4) involved depression-like behaviors secondary to other underlying disease conditions.

### Data extraction

Two authors (YH-W and YC-J) independently extracted data from the included studies using a predefined data extraction form. The following information was collected from each study: (1) first author and year of publication; (2) sample size, species, sex, body weight, and type of depression model; (3) dosage, route, and duration of ginsenoside administration; and (4) behavioral outcomes, including the sucrose preference test (SPT), forced swim test (FST), tail suspension test (TST), and open field test (OFT). Outcome data were extracted directly from tables and figures whenever available. When numerical values were not explicitly reported, they were estimated from graphical data using WebPlotDigitizer or requested from the corresponding author by email. For studies reporting multiple treatment arms, dose groups, or behavioral outcomes, each eligible comparison was extracted according to the reported experimental design, and the potential non-independence among comparisons was taken into account when interpreting the findings. Any discrepancies in data extraction were resolved through discussion between the two investigators, with adjudication by a third author (ZJ.S.) when necessary.

### Quality assessment

Risk of bias was independently assessed by two investigators (YH-W and ZJ-S) using the Systematic Review Center for Laboratory Animal Experimentation (SYRCLE) risk-of-bias tool for animal studies (Hooijmans et al., 2014). Disagreements were resolved through consultation with a third author (MF-D) (MF-D). The following domains were assessed: sequence generation, baseline characteristics, allocation concealment, random housing, blinding of caregivers and/or outcome assessors, random outcome assessment, incomplete outcome data, selective outcome reporting, and other potential sources of bias. Each domain was rated as having a low, high, or unclear risk of bias.

### Statistical analysis

Statistical analyses and figure generation were performed using RevMan 5.4.1 and Stata 14.0. The primary outcome was the SPT, and the secondary outcomes were the FST, TST, and OFT. In addition, an exploratory dose-stratified subgroup analysis was conducted for GRb1 to examine potential differences across dose ranges represented in the available literature. Treatment effects were summarized as standardized mean differences (SMDs) with 95% confidence intervals (CIs). Between-study heterogeneity was assessed using the I^2^ statistic, with I^2^ > 50% considered indicative of substantial heterogeneity. Given the expected methodological and biological diversity across animal species, depression models, dosing regimens, administration routes, and behavioral protocols, a random-effects model was used for pooled analyses. When multiple eligible treatment arms or doses from the same experiment contributed to the same outcome-specific pooled analysis, the corresponding comparisons were treated as separate effect sizes, including comparisons sharing a common control group. Outcomes were synthesized within their respective outcome-specific analyses. No formal statistical correction was applied for shared control groups or for potential within-study correlation among effect sizes. Consequently, residual statistical dependence may remain, and the uncertainty of some pooled estimates may have been underestimated. This issue is therefore considered a methodological limitation.

Interaction analyses were performed to explore whether pooled effects differed among ginsenoside components within the same behavioral domain. Because direct head-to-head comparisons among most ginsenosides were unavailable, these analyses were considered exploratory indirect comparisons and were not interpreted as evidence of comparative superiority. For studies contributing multiple intervention arms or dose groups, effect estimates were interpreted cautiously because comparisons sharing animals or control groups could not always be treated as statistically independent.

Publication bias was assessed by visual inspection of funnel plots when the number of independent studies was sufficient for meaningful interpretation. All tests were two-sided, and *p* < 0.05 was considered statistically significant.

## Results

### Study selection

The systematic database search identified 657 records. After removal of 342 duplicates, 315 unique records remained. Title and abstract screening excluded 261 records as irrelevant. The full texts of the remaining 54 articles were then assessed for eligibility, and 25 studies met the prespecified criteria and were included in the final analysis. The study selection process is summarized in the PRISMA flow diagram (Figure 1).

### Characteristics of included studies

All 25 included studies used male rodents, and the sample size per group generally ranged from 6 to 15 animals. With respect to animal strains, C57BL/6 mice were the most frequently used (11 studies), followed by ICR mice (7 studies), Wistar rats (3 studies), Sprague-Dawley rats (2 studies), and Kunming mice (1 study). One study did not report the strain of the experimental animals (Shi et al., 2022).

The methods used to establish depression models were heterogeneous and encompassed nine distinct modeling strategies. Among these, chronic unpredictable mild stress (CUMS), used in seven studies, was the most common paradigm. Other conceptually related stress-based models, including chronic mild stress (CMS), chronic unpredictable stress (CUS), and unpredictable mild stress (UMS), together constituted a major category of stress-induced depression models. Chronic restraint stress (CRS) and chronic social defeat stress (CSDS) were also represented. In addition, five studies used the lipopolysaccharide (LPS)-induced model, which is commonly used to mimic inflammation-related depressive-like phenotypes through activation of microglia and induction of neuroinflammation (Mariani et al., 2022; Yin et al., 2023). The reserpine model was based on monoamine depletion induced by this antihypertensive agent (Strawbridge et al., 2023). By contrast, the L-alpha-aminoadipic acid (L-AAA) model, established by stereotaxic injection into specific brain regions, represented a more specialized experimental approach (David et al., 2019).

Across the 25 included studies, the antidepressant-like effects of 10 ginsenoside monomers (GRb1, GRg1, GRg2, GRg3, GRe, GRc, GRd, GRh2, GRh4, and GRk1) as well as total ginsenosides were evaluated. Among these, GRb1 and GRg1 were the most extensively studied. Regarding administration, 20 studies used oral gavage, whereas five used intraperitoneal injection. Dose ranges, treatment durations, and evaluation criteria varied across studies, and not all studies included an active comparator drug (Li C. et al., 2024; Liang et al., 2022; Wang et al., 2022; Wang et al. 2021; Wang et al. 2025; Wang et al. 2023; Yu et al., 2024). In studies evaluating GRb1, doses below 35 mg/kg/day were used in four studies (Li et al., 2023; Liang et al., 2022; Wang et al., 2022; Zhang et al., 2021), whereas doses equal to or above 35 mg/kg/day were used in three studies (Jiang et al., 2021; Jiang et al., 2022; Li C. et al., 2024). In studies investigating GRg1, the selected doses were mainly 20 and 40 mg/kg/day (He et al., 2023; Li J. et al., 2022; Wang et al., 2021; Wang et al. 2025; Wang et al. 2023; Yu et al., 2024). Detailed characteristics of the included studies are presented in Table 1.

**TABLE 1:** Characteristics, intervention, and indices of the included studies.

Ginsenoside	Animals	Models	Intervention groups	Model groups	Positive control groups	Outcome (vs. model group)	Proposed mechanisms	Author, publication year
Rb1	Sprague-Dawley rats (Male, 215 ± 30 g, n = 6)	CUMS, 42 d	i.g., 42 d 10 mg/kg/d	Vehicle, 42 d	Escitalopram, i.g 10 mg/kg/d, 42 d	(1) SPT, *P* < 0.05 (2) FST, *P* < 0.05 (3) OFT (immobility time), *P* < 0.001	(1) Modulating mitochondrial autophagy (2) Suppressing astrocytic pyroptosis and neuroinflammation (3) Enhancing synaptic plasticity	Li et al. (2023) 37722244
Rb1	ICR mice (Male, 20 ± 2 g, n = 7–9)	CUMS, 42 d	i.g., 21 d 20 mg/kg/d	Vehicle, 21 d	-	(1) SPT, P < 0.05 (2) FST, P < 0.01 (3) TST, P < 0.01 (4) OFT (crossing number), P > 0.05 (5) OFT (total distance), P > 0.05 (6) OFT (central diatance), P > 0.05 (7) OFT (peripheral diatance), P > 0.05	Modulating hippocampal synaptic plasticity	Wang et al. (2022) 35600767
Rb1	C57BL/6 mice (Male, 21 ± 1 g, n = 15)	CRS, 21 d	i.g., 28 d 35 mg/kg/d	Vehicle, 28 d	-	(1) SPT, P < 0.001 (2) FST, P < 0.05 (3) OFT (crossing number), P > 0.05 (4) OFT (rearing number), P > 0.05	(1) Regulating TLR4/NF-κB/C3 pathway (2) Enhancing synaptic plasticity	Li C. et al. (2024) 39034364
Rb1	C57BL/6 mice (Male, 20–22 g, n = 10)	CSDS, 28 d	i.p., 28 d 35, 70 mg/kg/d	Vehicle, 28 d	IMI, i.p 15 mg/kg/d, 28 d	(1) FST (35 mg/kg/d: P < 0.05; 70 mg/kg/d: P < 0.05) (2) TST (35 mg/kg/d: P < 0.05; 70 mg/kg/d: P < 0.01) (3) OFT (total distance), 35 mg/kg/d: P > 0.05; 70 mg/kg/d: P > 0.05 (4) OFT (movement time), 35 mg/kg/d: P > 0.05; 70 mg/kg/d: P > 0.05 (5) SIT (35 mg/kg/d: P < 0.01; 70 mg/kg/d: P < 0.01)	(1) Suppressing inflammation (2) Inhibiting oxidative stress (3) Modulating SIRT1-NLRP3/Nrf2 pathway	Jiang et al. (2022) 35634375
Rb1	ICR mice (Male, 8-week-old, n = 12)	LPS, i.p., 1 d 1 mg/kg/d	i.g., 11 d 10, 20 mg/kg/d	Vehicle, 11 d	-	(1) FST (10 mg/kg/d: P < 0.05; 20 mg/kg/d: P < 0.01) (2) TST (10 mg/kg/d: P < 0.01; 20 mg/kg/d: P < 0.01)	(1) Suppressing inflammation (2) Improving impaired glucocorticoid receptor (3) Modulating MAPK/NF-κB pathway	Liang et al. (2022) 34537284
Rb1	C57BL/6J (Male, 7-8-week-old, n = 12)	CSDS, 38 d	i.g., 33 d 35, 70 mg/kg/d	Vehicle, 33 d	IMI, i.g 15 mg/kg/d, 33 d	(1) SPT (35 mg/kg/d: P < 0.01; 70 mg/kg/d: P < 0.01) (2) FST (35 mg/kg/d: P < 0.01; 70 mg/kg/d: P < 0.01) (3) SIT (SI ratio) (35 mg/kg/d: P < 0.01; 70 mg/kg/d: P < 0.01)	(1) Enhancing the BDNF signaling cascade (2) Upregulation of hippocampal neurogenesis	Jiang et al. (2021) 34658847
Rb1	C57BL/6J (Male, 18–22 g, n = 10)	CMS, 56 d	i.g., 28 d 20 mg/kg/d	Vehicle, 28 d	IMI, i.g 20 mg/kg/d, 28 d	(1) SPT, P < 0.001 (2) FST, P < 0.001 (3) TST, P < 0.001 (4) Locomotor activity, P > 0.05 (5) Body weight, P < 0.01	(1) Regulating PPARγ-mediated microglial activation (2) Improving hippocampus neurogenesis	Zhang et al. (2021) 34372875
Rg1	Sprague-Dawley rats (Male, 200–250 g, n ≥ 5)	CRS, 28 d	i.g., 28 d 20 mg/kg/d	Vehicle, 28 d	Escitalopram, i.g 1 mg/kg/d, 28 d	(1) SPT, P < 0.01 (2) FST, P < 0.01 (3) OFT (crossing number), P < 0.01 (4) OFT (rearing number), P < 0.001	(1) Suppressing microglial overactivation (2) Ameliorating mitochondrial dysfunction	Li J. et al. (2022) 35230663
Rg1	C57BL/6J mice (Male, 20 ± 1 g, n = 12)	UMS, 7 d	i.g., 14 d 20, 40 mg/kg/d	Vehicle, 14 d	-	(1) FST (20 mg/kg/d: P < 0.01; 40 mg/kg/d: P < 0.001) (2) TST (20 mg/kg/d: P < 0.05; 40 mg/kg/d: P < 0.01) (3) MBT (20 mg/kg/d: P < 0.05; 40 mg/kg/d: P < 0.001)	Regulating gut-brain axis	Yu et al. (2024) 39515104
Rg1	Wistar rats (Male, 275 ± 25 g, n = 9)	CUS, 72 d	i.g., 72 d 40 mg/kg/d	Vehicle, 72 d	-	(1) FST, P < 0.001 (2) SPT, P < 0.001 (3) OFT (total distance), P < 0.001 (4) OFT (crossing number), P < 0.001 (5) OFT (rearing number), P < 0.05	(1) Regulating Cx43-YAP axis (2) Inhibiting neuroinflammation	Wang et al. (2025) 40055527
Rg1	Wistar rats (Male, 170 ± 10 g, n = 12)	LPS, i.p., 14 d 0.5 mg/kg/d	i.g., 14 d 40 mg/kg/d	Vehicle, 14 d	-	(1) SPT, P < 0.001 (2) FST, P < 0.01 (3) OFT (total distance), P > 0.05 (4) OFT (time in the central area), P < 0.05 (5) EPM (open arm entry number), P < 0.05 (6) EPM (open arm times), P < 0.05	(1) Suppressing glial hyperactivation and neuroinflammation (2) Ameliorating synaptic plasticity dysfunction	Wang et al. (2023) 37464840
Rg1	Wistar rats (Male, 250–300 g, n = 6)	CUS, 28 d	i.p., 31 d 40 mg/kg	Vehicle, 31 d	-	(1) SPT, P < 0.01 (2) OFT (total distance), P < 0.05 (3) OFT (crossing number), P < 0.05	(1) Suppressing Cx43 ubiquitination-mediated degradation (2) Alleviating neuroinflammation	Wang et al. (2021) 34421601
Rg1	C57BL/6J mice (Male, 18–22 g, n = 8)	CMS, 42 d	i.p., 21 d 20 mg/kg	Vehicle, 21 d	IMI, i.p 20 mg/kg/d, 21 d	(1) SPT, P < 0.001 (2) FST, P < 0.001 (3) TST, P < 0.01	(1) Suppressing activation of NLRP3 inflammasome and pro-inflammatory microglia (2) Rescuing neurogenesis	He et al. (2023) 37863415
Rg2	C57BL/6J mice (Male, 8-week-old, n = 10)	CMS, 42 d	i.p., 14 d 10,20 mg/kg	Vehicle, 14 d	Fluoxetine, i.p 10 mg/kg/d, 14 d	(1) SPT (10 mg/kg/d: P < 0.05; 20 mg/kg/d: P < 0.01) (2) FST (10 mg/kg/d: P < 0.05; 20 mg/kg/d: P < 0.01) (3) TST (10 mg/kg/d: P < 0.05; 20 mg/kg/d: P < 0.01)	Regulating BDNF-TrkB axis	Ren et al. (2017) 28842305
Rg3	ICR mice (Male, 8-week-old, n = 10)	LPS, i.p., 1 d 0.83 mg/kg/d	i.g., 3 days (twice daily) 20,40 mg/kg	Vehicle, 3 d	Minocycline, i.p 50 mg/kg, 2 d	(1) FST (20 mg/kg/d: P < 0.05; 40 mg/kg/d: P < 0.01) (2) TST (20 mg/kg/d: P < 0.05; 40 mg/kg/d: P < 0.01)	Ameliorating immune activation	Kang et al. (2017) 28762741
Rg3	C57BL/6J mice (Male, 18–22 g, n = 10)	CMS, 56 d	i.g., 28 d 50,100 and 150 mg/kg	Vehicle, 28 d	Fluoxetine, i.g 10 mg/kg/d, 28 d	(1) SPT (50 mg/kg/d: P > 0.05; 100 mg/kg/d: P > 0.05; 150 mg/kg/d: P < 0.05) (2) FST (50 mg/kg/d: P > 0.05; 100 mg/kg/d: P > 0.05; 150 mg/kg/d: P > 0.05) (3) TST (50 mg/kg/d: P > 0.05; 100 mg/kg/d: P < 0.05; 150 mg/kg/d: P > 0.05)	Regulating CREB/BDNF pathway	Zhang et al. (2017) 28461003
Re	ICR mice (Male, 18–20 g, n = 9)	Reserpine, 3 d 1 mg/kg/d	i.g., 7 d 10, 30 and 90 mg/kg/d	Vehicle, 7 d	Fluoxetine, i.g 10 mg/kg/d, 7 d	(1) FST (10 mg/kg/d: P > 0.05; 30 mg/kg/d: P < 0.05; 90 mg/kg/d: P > 0.05) (2) TST (10 mg/kg/d: P > 0.05; 30 mg/kg/d: P < 0.05; 90 mg/kg/d: P > 0.05) (3) OFT (crossing number), 10 mg/kg/d: P > 0.05; 30 mg/kg/d: P > 0.05; 90 mg/kg/d: P > 0.05) (5) OFT (rearing number), 10 mg/kg/d: P > 0.05; 30 mg/kg/d: P > 0.05; 90 mg/kg/d: P > 0.05)	(1) Inhibiting neuroinflammation and oxidative stress (2) Ameliorating synaptic plasticity (3) Modulating the BDNF/TrkB/ERK/CREB pathway	Chen et al. (2024) 39186472
Re	C57BL/6J mice (Male, 6-week-old, n = 8)	CUMS, 28 d	i.g., 14 d 20, 40 mg/kg/d	Vehicle, 14 d	Fluoxetine, i.g 10 mg/kg/d, 14 d	(1) SPT (20 mg/kg/d: P < 0.05; 40 mg/kg/d: P < 0.05) (2) TST (20 mg/kg/d: P < 0.05; 40 mg/kg/d: P < 0.05) (3) OFT (total distance), 20 mg/kg/d: P < 0.05; 40 mg/kg/d: P < 0.05 (4) OFT (rearing number), 20 mg/kg/d: P < 0.05; 40 mg/kg/d: P < 0.05 (5) OFT (time in the central area), 20 mg/kg/d: P < 0.05; 40 mg/kg/d: P < 0.05	(1) Promoting PINK1-Mediated Mitophagy (2) Inhibiting neuroinflammation	Liu et al. (2025) 40298124
Rc	C57BL/6J mice (Male, 19–21 g, n = 9)	L-AAA (100 μg/mL)	i.g., 7 d 20 mg/kg/d	Vehicle, 7 d	Imipramine, i.g 1 mg/mL, 7 d	(1) FST, P < 0.001 (2) TST, P < 0.001 (3) OFT (total distance), P > 0.05	(1) Ameliorating astrocytic ablation (2) Curbing neuroinflammation	Kwon et al. (2024) 39273621
Rd	ICR mice (Male, 20–23 g, n = 8)	LPS, 1 d 0.83 mg/kg/d	i.g., 14 d 4, 12 and 40 mg/kg/d	Vehicle, 14 d	Fluoxetine, i.g 20 mg/kg/d, 14 d	(1) SPT (4 mg/kg/d: P > 0.05; 12 mg/kg/d: P > 0.05; 40 mg/kg/d: P < 0.05) (2) FST (4 mg/kg/d: P > 0.05; 12 mg/kg/d: P > 0.05; 40 mg/kg/d: P < 0.01) (3) TST (4 mg/kg/d: P > 0.05; 12 mg/kg/d: P < 0.05; 40 mg/kg/d: P < 0.01) (4) OFT (crossing number), 4 mg/kg/d: P > 0.05; 12 mg/kg/d: P > 0.05; 40 mg/kg/d: P > 0.05 (5) OFT (rearing number), 4 mg/kg/d: P > 0.05; 12 mg/kg/d: P > 0.05; 40 mg/kg/d: P > 0.05	(1) Suppressing neuroinflammation by modulating TLR4-PI3K-NF-κB-JMJD3 pathway (2) Ameliorating synaptic plasticity	Chen et al. (2025) 40836407
Rd	ICR mice (Male, 18–22 g, n = 10)	CUMS, 49 d	i.g., 21 d 10, 20 mg/kg/d	Vehicle, 21 d	Paroxetine, i.p 10 mg/kg/d, 21 d	(1) SPT (10 mg/kg/d: P < 0.05; 20 mg/kg/d: P < 0.01) (2) OFT (total distance), 10 mg/kg/d: P < 0.05; 20 mg/kg/d: P < 0.01	(1) Regulating HIF-1α pathway (2) Ameliorating synaptic plasticity	Li Y. et al. (2022) 35370402
Rh2	Adult mice (Male, 20–22 g, n = 10)	CUMS, 42 d	i.p., 14 d 10, 20 mg/kg/d	Vehicle, 14 d	Fluoxetine, i.p 20 mg/kg/d, 14 d	(1) SPT (10 mg/kg/d: P > 0.05; 20 mg/kg/d: P > 0.05) (2) FST (10 mg/kg/d: P < 0.01; 20 mg/kg/d: P < 0.01) (3) TST (10 mg/kg/d: P < 0.01; 20 mg/kg/d: P < 0.01)	(1) Modulating BDNF-TrkB pathway (2) Promoting neurogenesis	Shi et al. (2022) 35848938
Rh4	C57BL/6J mice (Male, 20–25 g, n = 10)	CUMS, 63 d	i.g., 42 d 20, 40 mg/kg/d	Vehicle, 42 d	Imipramine, i.g., 42 d 15 mg/kg/d	(1) SPT (20 mg/kg/d: P < 0.01; 40 mg/kg/d: P < 0.001) (2) FST (20 mg/kg/d: P < 0.01; 40 mg/kg/d: P < 0.001) (3) TST (20 mg/kg/d: P < 0.01; 40 mg/kg/d: P < 0.001)	(1) Alleviating neuronal damage and HPA axis dysregulation (2) Suppressing inflammation (3) Improving intestinal flora and short-chain fatty-acids (4) Modulating LPS/NLRP3/caspase-1/IL-1β pathway	Shao et al. (2023) 36283312
Rk1	ICR mice (Male, 18–22 g, n = 8)	LPS, 1 d 0.83 mg/kg/d	i.g., 7 d 5, 10 and 20 mg/kg/d	Vehicle, 42 d	Fluoxetine, i.g 20 mg/kg/d, 7 d	(1) FST (5 mg/kg/d: P < 0.05; 10 mg/kg/d: P < 0.01; 20 mg/kg/d: P < 0.01) (2) TST (5 mg/kg/d: P < 0.05; 10 mg/kg/d: P < 0.01; 20 mg/kg/d: P < 0.001)	(1) Antioxidant (2) Inhibiting inflammation (3) Modulating BDNF-TrkB pathway	Li et al. (2020) 32768191
Ginsenosides	Kunming mice (Male, 18–22 g, n = 12)	CUMS, 42 d	i.g., 21 d 50, 100 and 200 mg/kg/d	Vehicle, 21 d	Venlafaxine, i.g 10 mg/kg/d, 21 d	(1) SPT (50 mg/kg/d: P < 0.001; 100 mg/kg/d: P < 0.001; 200 mg/kg/d: P < 0.05) (2) OFT (total distance), 50 mg/kg/d: P > 0.05; 100 mg/kg/d: P < 0.001; 200 mg/kg/d: P > 0.05 (3) OFT (rearing number), 50 mg/kg/d: P > 0.05; 100 mg/kg/d: P < 0.001; 200 mg/kg/d: P > 0.05	Modulating HPA function by inhibiting FKBP51 on glucocorticoid receptor	Li H. et al. (2024) 39278841

### Antidepressant mechanisms of ginsenoside monomers and total ginsenosides

The included studies suggested that ginsenoside monomers may ameliorate depression-like behaviors through multiple targets and pathways, with evidence of neuroprotective activity. Among the investigated compounds, GRb1 was reported to improve stress-related abnormalities in neuroplasticity, neuroinflammation, oxidative stress, and neurotransmitter homeostasis through coordinated regulation of several signaling pathways. Available studies indicated that GRb1 promoted adult hippocampal neurogenesis, increased the number of newborn neurons, and improved synaptic structure and function, including dendritic spine density, long-term potentiation (LTP), and synaptic protein expression, thereby attenuating stress-induced synaptic dysfunction (Li C. et al., 2024; Zhang et al., 2021). Its neurotrophic effects were mainly linked to activation of BDNF signaling, and the antidepressant-like effect of GRb1 was abolished after TrkB inhibition, supporting an important role for the BDNF/TrkB axis (Jiang et al., 2021). GRb1 was also reported to suppress microglial and astrocytic activation and to reduce the expression of pro-inflammatory cytokines, including TNF-alpha, IL-1beta, and IL-18, partly through inhibition of NLRP3 inflammasome activation and stimulation of the PPARgamma pathway (Jiang et al., 2022; Zhang et al., 2021). In addition, GRb1 attenuated oxidative damage by increasing hippocampal superoxide dismutase (SOD) and catalase (CAT) activity, reducing lipid peroxidation (LPO), and activating the SIRT1/Nrf2/HO-1 antioxidant pathway (Jiang et al., 2022). More recent work further suggested that GRb1 may directly bind to TLR4, inhibit its conformational activation, block the TLR4/NF-kappaB/C3 signaling cascade, and reduce complement C3-mediated excessive synaptic pruning, thereby protecting hippocampal synaptic integrity (Li C. et al., 2024).

Compared with GRb1, the reported mechanisms of GRg1 were more focused on neuroinflammation and gut-brain axis regulation. GRg1 significantly attenuated microglial activation and neuroinflammatory responses, mainly through suppression of NLRP3 inflammasome activation and downregulation of pro-inflammatory cytokines such as IL-1beta and IL-18 (He et al., 2023). At the molecular level, GRg1 was shown to improve microglial mitochondrial dysfunction through the long non-coding RNA GAS5/EZH2/SOCS3/NRF2 signaling axis (Li J. et al., 2022). It was also reported to bind directly to connexin 43 (Cx43), inhibit its ubiquitination, and regulate the Cx43-YAP pathway, thereby reversing YAP nuclear translocation and ameliorating neuroinflammation and synaptic dysfunction (Wang et al., 2021; Wang et al., 2025). In one study, GRg1 also acted synergistically with voluntary wheel running to further reduce CD45, CD11b, and IL-1beta protein levels in the hippocampus and prefrontal cortex (Wang et al., 2023). In addition, GRg1 was reported to modulate gut microbiota composition, particularly by promoting the growth of L. murinus, thereby influencing gut-brain axis signaling and depressive-like behaviors (Yu et al., 2024).

The antidepressant-like effects of GRg2 and GRg3 were mainly associated with regulation of neurotrophic factors and inflammatory responses. GRg2 significantly improved depressive-like behaviors, and this effect appeared to depend largely on activation of hippocampal BDNF/TrkB signaling (Ren et al., 2017). Similarly, GRg3 exerted antidepressant-like effects by promoting BDNF expression (Zhang et al., 2017). As a characteristic constituent of red ginseng, GRg3 was also reported to modulate the tryptophan-kynurenine pathway, thereby alleviating LPS-induced systemic and neuroinflammation, suppressing excessive microglial activation, and improving inflammation-related depressive-like behaviors (Kang et al., 2017).

GRh2 and GRh4 appeared to act through distinct molecular pathways. In the CUMS model, the antidepressant-like effect of GRh2 depended on activation of hippocampal BDNF/CREB signaling and was abolished after TrkB inhibition (Shi et al., 2022). By contrast, GRh4 primarily attenuated depressive-like behaviors by reducing oxidative stress in hippocampal neurons, markedly decreasing excessive CUMS-induced ROS production while also suppressing abnormal activation of microglia and astrocytes (Shao et al., 2023).

Other ginsenoside monomers were also reported to exert antidepressant-like effects through distinct mechanisms. GRk1 significantly improved LPS-induced depressive-like behaviors by promoting BDNF expression and suppressing the release of pro-inflammatory cytokines including TNF-alpha, IL-1beta, and IL-6 (Li et al., 2020). GRe activated mitophagy through upregulation of PINK1, inhibited H2O2-induced ROS accumulation and inflammatory responses, and modulated the p-CREB/TrkB/AKT signaling axis to enhance neuroplasticity (Chen et al., 2024; Liu et al., 2025). GRd alleviated neuroinflammation by blocking the TLR4/PI3K/NF-kappaB/JMJD3 pathway and improved cerebral blood perfusion through modulation of the HIF-1alpha/VEGF pathway (Chen et al., 2025; Li Y. et al., 2022). Some studies also suggested that certain ginsenosides may enhance glucocorticoid receptor sensitivity by inhibiting FKBP51 and thereby contribute to restoration of HPA-axis homeostasis (Li H. et al., 2024), whereas GRc may mitigate neuroinflammation through regulation of astrocyte function (Kwon et al., 2024). Taken together, these findings suggest that the antidepressant-related effects of ginsenosides are mechanistically multidimensional and may involve convergent regulation of neuroplasticity, inflammation, oxidative stress, and neuroendocrine signaling.

### Quality assessment of the included studies

Methodological quality was assessed using the SYRCLE risk-of-bias tool for animal studies. Among the 25 included studies, only one showed an unclear risk of bias for sequence generation. For baseline characteristics, 19 studies were judged to be at low risk of bias, whereas the remaining six were rated as unclear. No study provided sufficient detail on allocation concealment. Owing to random housing procedures, only one study was judged to be at high risk of performance bias. Assessment of blinding-related domains and reporting bias was frequently limited by incomplete reporting, which is common in preclinical animal studies. A summary of the risk-of-bias assessment for each included study is shown in Figure 2.

**FIGURE 2:**
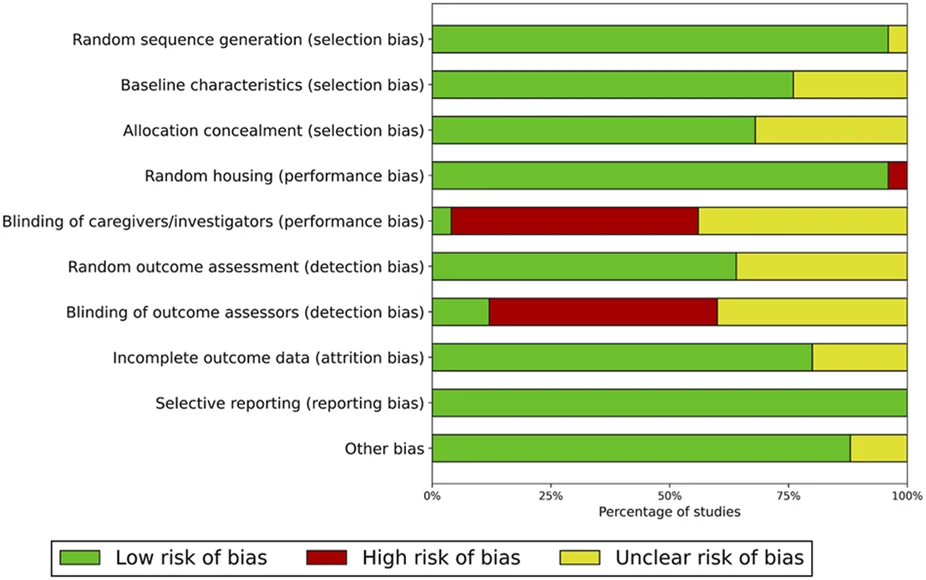
Risk of bias in the included studies.

### The ameliorative effect of ginsenosides on anhedonia-like behavior

The sucrose preference test (SPT) is a widely used behavioral paradigm for assessing anhedonia, a core feature of depressive-like phenotypes in animal models. An increase in sucrose preference is generally interpreted as an improvement in hedonic function following antidepressant intervention (Primo et al., 2023). For this reason, the SPT was designated as the primary outcome of the present study.

Eighteen of the 25 included studies reported SPT data, contributing 30 direct or indirect comparisons and a total of 575 animals. The pooled analysis showed that, compared with saline-treated model controls, ginsenoside monomers and total ginsenosides significantly reversed the decline in sucrose preference (SMD = 3.20, 95% CI = 2.61–3.80; *p* < 0.01), supporting an overall beneficial effect on anhedonia-like behavior (Figure 3).

**FIGURE 3:**
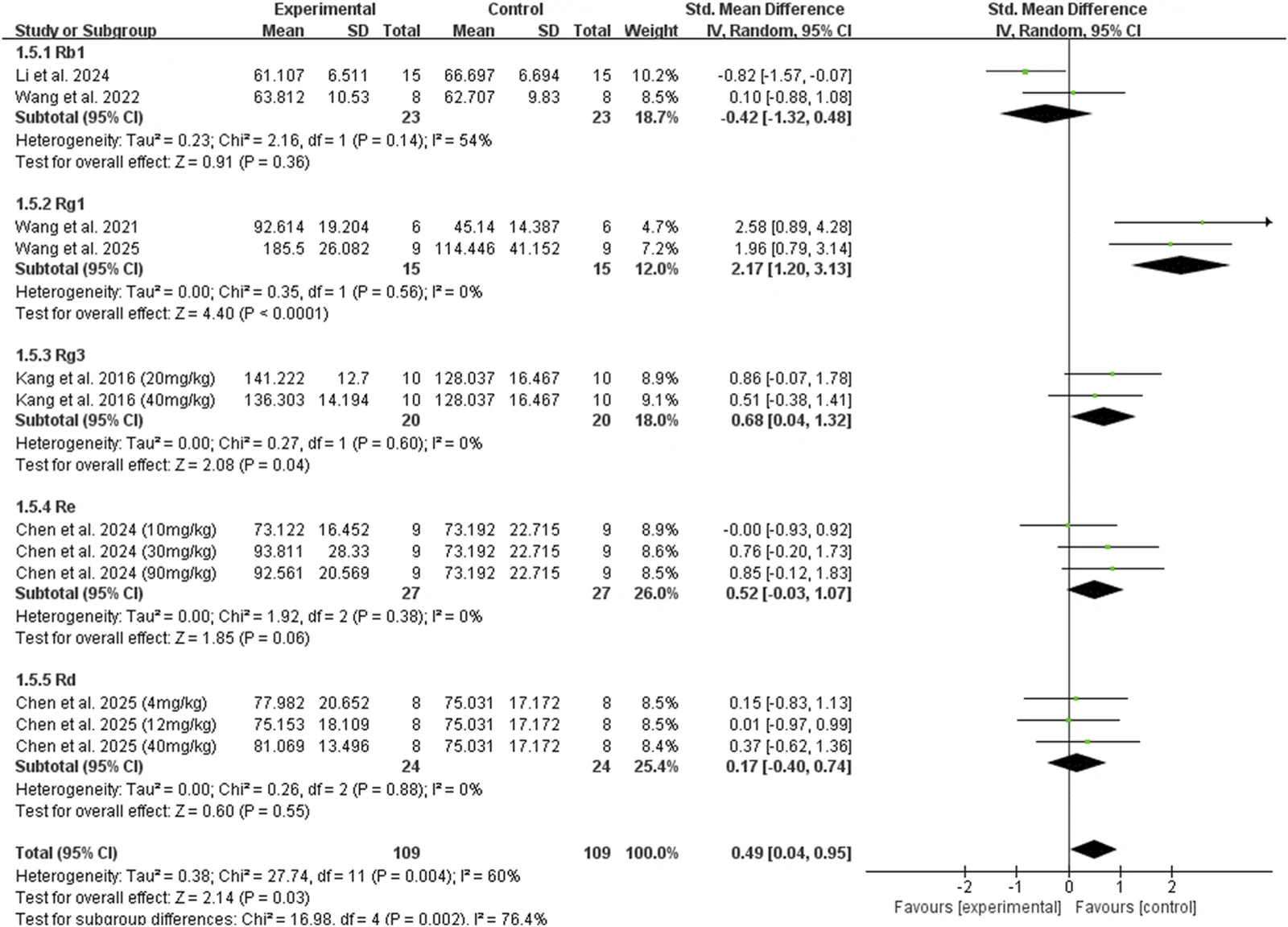
Forest plot for the effect of ginsenoside monomers and total ginsenosides on the SPT. In this representation, an effect size with a 95% CI greater than 0—indicated by a horizontal line that lies entirely to the right of the vertical null line without intersecting it—signifies a significantly higher outcome rate in the treatment group compared to the model group. The meta-analysis demonstrated that the treatment group effectively increased sucrose solution consumption in animals (SMD: 3.20, 95% CI: 2.61–3.80, Z = 10.52, *P* < 0.00001). However, significant heterogeneity was observed across the included studies (I^2^ = 81%, *P* < 0.00001). And the confidence intervals of three individual studies intersected the null line.

Among the evaluated interventions, GRb1 showed the largest pooled effect size in the available SPT dataset. Compared with saline-treated model controls, GRb1 significantly increased sucrose consumption or preference in experimental animals (SMD = 4.83, 95% CI: 3.17–6.49, *p* < 0.01) (Figure 3). However, this finding should be interpreted as indirect comparative evidence rather than a definitive ranking of efficacy. To further explore differences among monomers, we performed an interaction analysis using a matrix-style league table. This analysis suggested that the pooled effect associated with GRb1 was significantly larger than those of GRg3 (*p* for interaction = 0.003), GRe (*p* for interaction = 0.0004), and GRd (*p* for interaction = 0.002) (Table 2). These comparisons indicate that different ginsenosides may vary in their apparent effects on anhedonia-related behavior, although the results should be interpreted cautiously given the indirect nature of the comparisons and the heterogeneity of the included studies.

**TABLE 2:** Analysis of the interaction between ginsenoside monomers and total ginsenosides in the SPT.

Rb1	Rg1	Rg2	Rg3	Re	Rd	Rh2	Rh4	Ginsenosides
0.6	Rg1	​	​	​	​	​	​	​
0.22	0.32	Rg2	​	​	​	​	​	​
0.003	0.001	0.08	Rg3	​	​	​	​	​
0.0004	<0.01	0.01	0.22	Re	​	​	​	​
0.002	0.0004	0.05	0.53	0.68	Rd	​	​	​
0.14	0.19	0.62	0.47	0.18	0.3	Rh2	​	​
0.42	0.65	0.58	0.006	0.0006	0.006	0.34	Rh4	​
0.35	0.5	0.98	0.22	0.07	0.14	0.66	0.71	Ginsenosides

### The ameliorative effects of ginsenosides on behavioral despair

The FST and TST are classic paradigms used to assess behavioral despair in rodent models, and reduced immobility time is commonly interpreted as an antidepressant-like effect (Cryan et al., 2005; Molendijk and de Kloet, 2019). In the present analyses, immobility time was used as the outcome measure and summarized as the standardized mean difference (SMD). Overall, compared with saline-treated model controls, ginsenoside monomers and total ginsenosides showed significant activity in both the FST (SMD = −2.21, 95% CI: −2.66 to −1.76; *p* < 0.01) and the TST (SMD = −2.07, 95% CI: −2.45 to −1.68; *p* < 0.01).

Among the 25 included studies, 20 reported FST data, contributing 36 comparisons and 692 animals. Most evaluated ginsenoside monomers significantly reduced immobility time relative to saline-treated model controls. GRh2 showed the largest pooled effect size (SMD = −4.55, 95% CI: −5.82 to −3.27; *p* < 0.01), followed by GRb1 (SMD = −2.92, 95% CI: −3.92 to −1.93; *p* < 0.01) (Figure 4). Interaction analysis provided further indirect comparisons among monomers. The pooled effect associated with GRh2 was significantly larger than those of GRg3 (*p* for interaction <0.01), GRe (*p* for interaction <0.01), and GRd (*p* for interaction <0.01), and showed a borderline difference versus GRg2 (*p* for interaction = 0.08). GRb1 also showed significantly larger pooled effects than GRg3 (*p* for interaction = 0.009), GRe (*p* for interaction = 0.0001), and GRd (*p* for interaction = 0.002). By contrast, the indirect comparison between GRh2 and GRb1 did not demonstrate a clear statistically robust difference (*p* = 0.05) (Table 3). Overall, these findings suggest that GRh2 and GRb1 were among the compounds associated with comparatively large effects in the FST dataset, while not supporting a definitive efficacy hierarchy.

**FIGURE 4:**
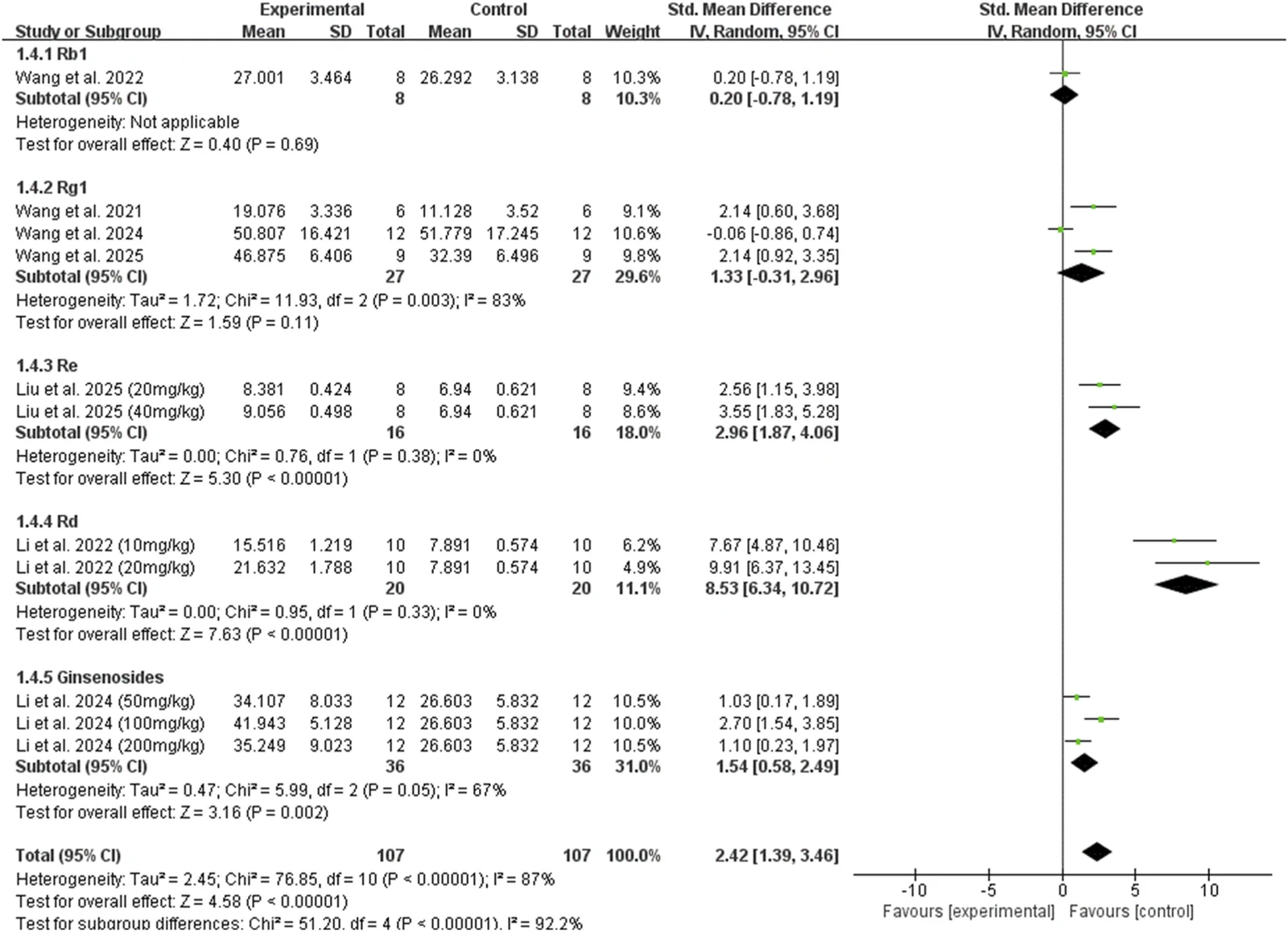
Forest plot for the effect of ginsenoside monomers and total ginsenosides on the FST. In this representation, an effect size with a 95% CI lower than 0—indicated by a horizontal line that lies entirely to the left of the vertical null line without intersecting it—signifies a significantly higher outcome rate in the treatment group compared to the model group. The meta-analysis demonstrated that the treatment group effectively significantly reduced the immobility time in model animals (SMD: −2.21, 95% CI: −2.66 to −1.76, Z = 9.72, *P* < 0.00001). However, significant heterogeneity was observed across the included studies (I^2^ = 79%, *P* < 0.00001). And the confidence intervals of five individual studies intersected the null line.

**TABLE 3:** Analysis of the interaction between ginsenoside monomers and total ginsenosides in the FST.

Rb1	Rg1	Rg2	Rg3	Re	Rd	Rh2	Rh4	Rk1
0.69	Rg1	​	​	​	​	​	​	​
0.97	0.69	Rg2	​	​	​	​	​	​
0.009	0.08	0.02	Rg3	​	​	​	​	​
0.0001	0.003	0.0007	0.05	Re	​	​	​	​
0.002	0.03	0.007	0.38	0.31	Rd	​	​	​
0.05	0.03	0.08	<0.01	<0.01	<0.01	Rh2	​	​
0.58	0.93	0.6	0.05	0.001	0.01	0.01	Rh4	​
0.3	0.64	0.34	0.08	0.001	0.02	0.003	0.66	Rk1

Fifteen studies reported TST data. In this analysis, GRh2 (SMD = −3.83, 95% CI: −4.96 to −2.70; *p* < 0.01), GRh4 (SMD = −3.68, 95% CI: −6.26 to −1.11; *p* < 0.01), and GRg2 (SMD = −3.56, 95% CI: −5.14 to −1.97; *p* < 0.01) showed relatively large pooled effects (Figure 5). Interaction analysis again suggested a more complex pattern of apparent differences across compounds. The pooled effect associated with GRh2 was significantly larger than those of GRg1 (*p* for interaction <0.0001), GRd (*p* for interaction <0.01), and GRk1 (*p* for interaction = 0.004). However, comparisons between GRb1 and GRh4 (*p* for interaction = 0.35) and between GRb1 and GRg2 (*p* for interaction = 0.20) did not show clear evidence of a difference, although the comparison between GRh4 and GRb1 approached nominal significance (*p* for interaction = 0.05). A significant difference was also observed between GRg2 and GRg1 (*p* = 0.009). In addition, the relative position of GRd in the TST differed from that observed in the SPT, with smaller pooled effects than GRb1 (*p* = 0.01) and GRh2 (*p* < 0.01) (Table 4). Taken together, these indirect comparisons suggest that the apparent effects of different ginsenosides in the TST may be behavior-specific and should not be assumed to mirror their profiles in the FST or SPT.

**FIGURE 5:**
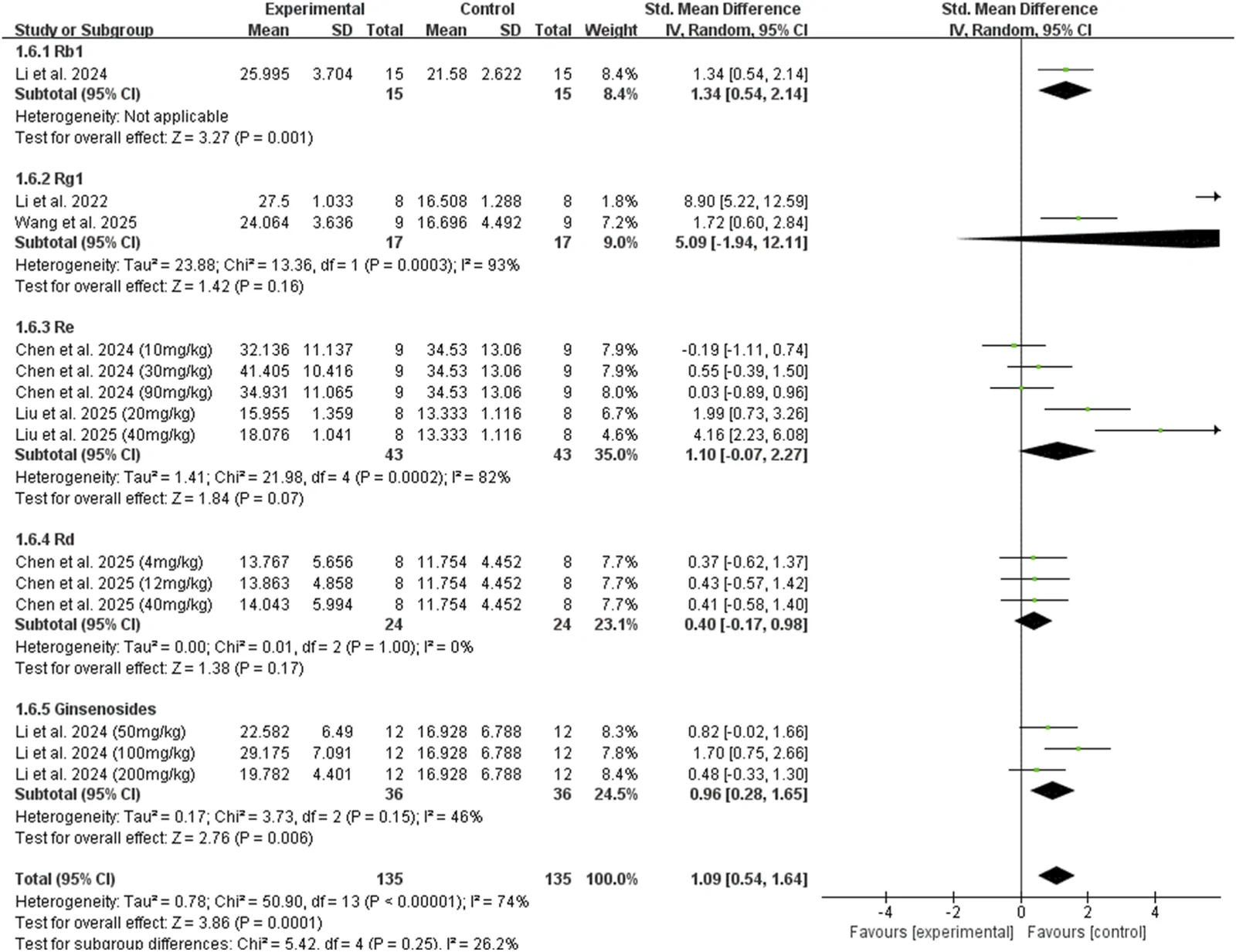
Forest plot for the effect of ginsenoside monomers and total ginsenosides on the TST. In this representation, an effect size with a 95% CI lower than 0—indicated by a horizontal line that lies entirely to the left of the vertical null line without intersecting it—signifies a significantly higher outcome rate in the treatment group compared to the model group. The meta-analysis demonstrated that the treatment group effectively significantly reduced the immobility time in model animals (SMD: −2.07, 95% CI: −2.45 to −1.68, Z = 10.44, *P* < 0.00001). However, significant heterogeneity was observed across the included studies (I^2^ = 69%, *P* < 0.00001). And the confidence intervals of three individual studies intersected the null line.

**TABLE 4:** Analysis of the interaction between ginsenoside monomers and total ginsenosides in the TST.

Rb1	Rg1	Rg2	Rg3	Re	Rd	Rh2	Rh4	Rk1
0.05	Rg1	​	​	​	​	​	​	​
0.2	0.009	Rg2	​	​	​	​	​	​
0.34	0.28	0.05	Rg3	​	​	​	​	​
0.33	0.57	0.05	0.82	Re	​	​	​	​
0.01	0.44	0.003	0.08	0.28	Rd	​	​	​
0.05	0.0001	0.78	0.003	0.007	<0.01	Rh2	​	​
0.35	0.05	0.93	0.17	0.16	0.04	0.92	Rh4	​
0.39	0.23	0.06	0.91	0.75	0.06	0.004	0.19	Rk1

### The ameliorative effects of ginsenosides on various parameters in the OFT

The OFT is commonly used to assess spontaneous locomotor activity, exploratory behavior, and anxiety-related responses in rodents, typically through multiple parameters including total distance traveled, number of crossings, and number of rearings (Cevik et al., 2025; Prut and Belzung, 2003). Because these parameters reflect partially distinct behavioral dimensions, separate meta-analyses were conducted for each outcome.

For total distance traveled, 7 studies comprising 11 comparisons and 214 animals were included. Compared with saline-treated model controls, ginsenoside monomers significantly increased total movement distance (SMD = 2.42, 95% CI: 1.39–3.46; *p* < 0.01). Among the evaluated compounds, GRd showed the largest pooled effect size (SMD = 8.53, 95% CI: 6.34–10.72; *p* < 0.01) (Figure 6). By contrast, neither GRb1 (SMD = 0.20, 95% CI: −0.78 to 1.19; *p* = 0.69) nor GRg1 (SMD = 1.33, 95% CI: −0.31 to 2.96; *p* = 0.11) showed a statistically significant increase in this parameter.

**FIGURE 6:**
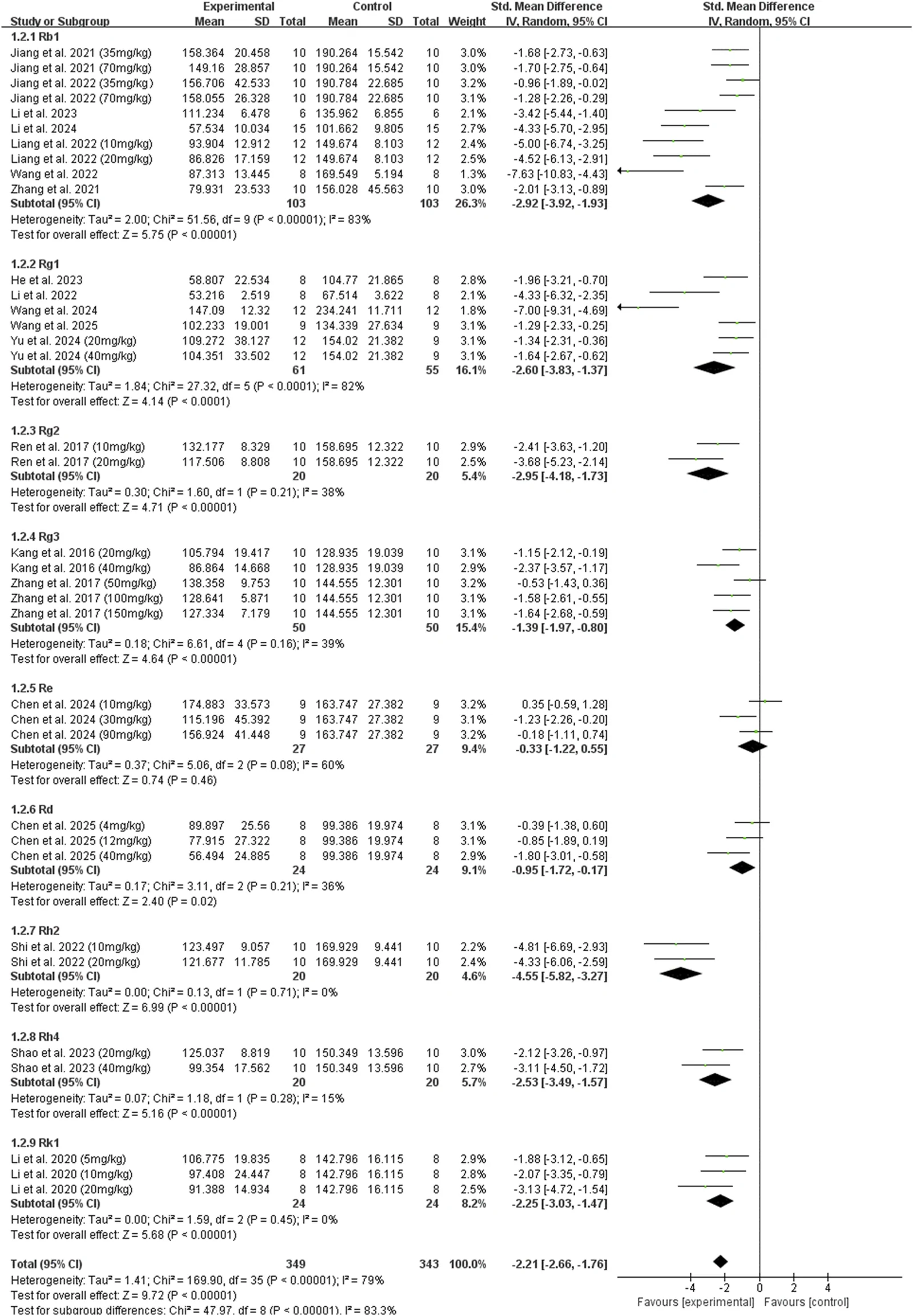
Forest plot for the effect of ginsenoside monomers and total ginsenosides on total distance in the OFT. In this representation, an effect size with a 95% CI greater than 0—indicated by a horizontal line that lies entirely to the right of the vertical null line without intersecting it—signifies a significantly higher outcome rate in the treatment group compared to the model group. The meta-analysis demonstrated that most treatment groups significantly increased the total distance in model animals (SMD: 2.42, 95% CI: 1.39–3.46, Z = 4.58, *P* < 0.00001). Significant heterogeneity was observed across the included studies (I^2^ = 87%, *P* < 0.00001).

For the number of crossings, 7 studies comprising 12 comparisons and 218 animals were included. The pooled results indicated that ginsenoside monomers increased the number of crossings relative to saline-treated model controls (SMD = 0.49, 95% CI: 0.04–0.95; *p* = 0.03). GRg1 showed the largest pooled effect on this parameter (SMD = 2.17, 95% CI: 1.20–3.13; *p* < 0.01) (Figure 7). The other evaluated monomers did not show comparably clear effects on horizontal activity.

**FIGURE 7:**
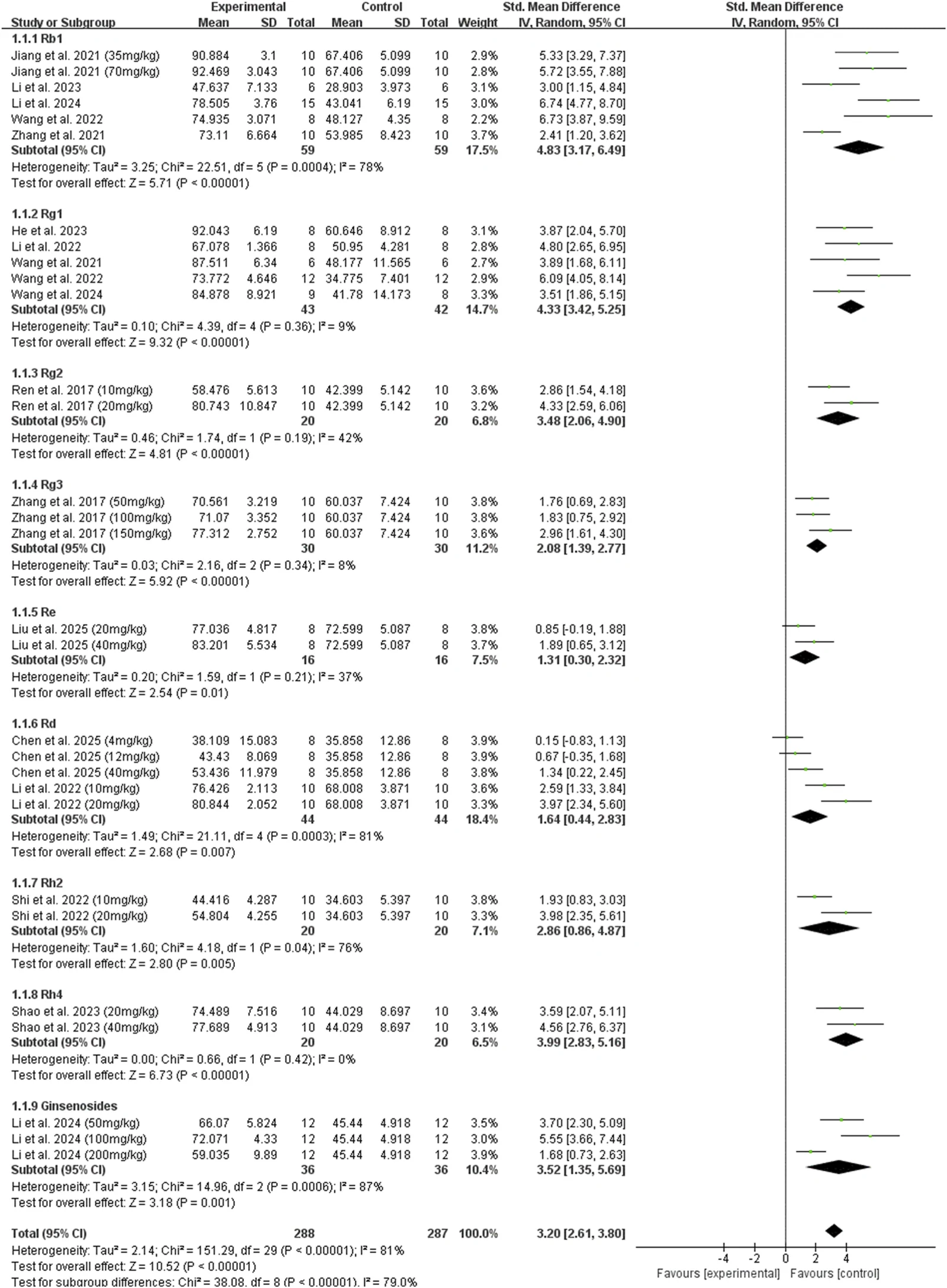
Forest plot for the effect of ginsenoside monomers and total ginsenosides on the number of crossings in the OFT. In this representation, an effect size with a 95% CI greater than 0—indicated by a horizontal line that lies entirely to the right of the vertical null line without intersecting it—signifies a significantly higher outcome rate in the treatment group compared to the model group. The meta-analysis demonstrated that most treatment groups significantly increased the number of crossings in model animals (SMD: 0.49, 95% CI: 0.04–0.95, Z = 2.14, *P* = 0.03). Significant heterogeneity was observed across the included studies (I^2^ = 60%, *P* = 0.004).

For the number of rearings, 7 studies comprising 14 comparisons and 270 animals were included. Overall, ginsenoside monomers increased rearing behavior compared with saline-treated model controls (SMD = 1.09, 95% CI: 0.54–1.64; *p* < 0.01). Although GRg1 showed a numerically large effect estimate (SMD = 5.09, 95% CI: −1.94 to 12.11; *p* = 0.16), the confidence interval was wide and the result was not statistically significant, indicating substantial uncertainty. GRb1 was associated with a significant increase in rearing behavior (SMD = 2.16, 95% CI: 1.03–3.29; *p* < 0.01) (Figure 8). The remaining evaluated monomers did not show strong or consistent effects on this specific parameter.

**FIGURE 8:**
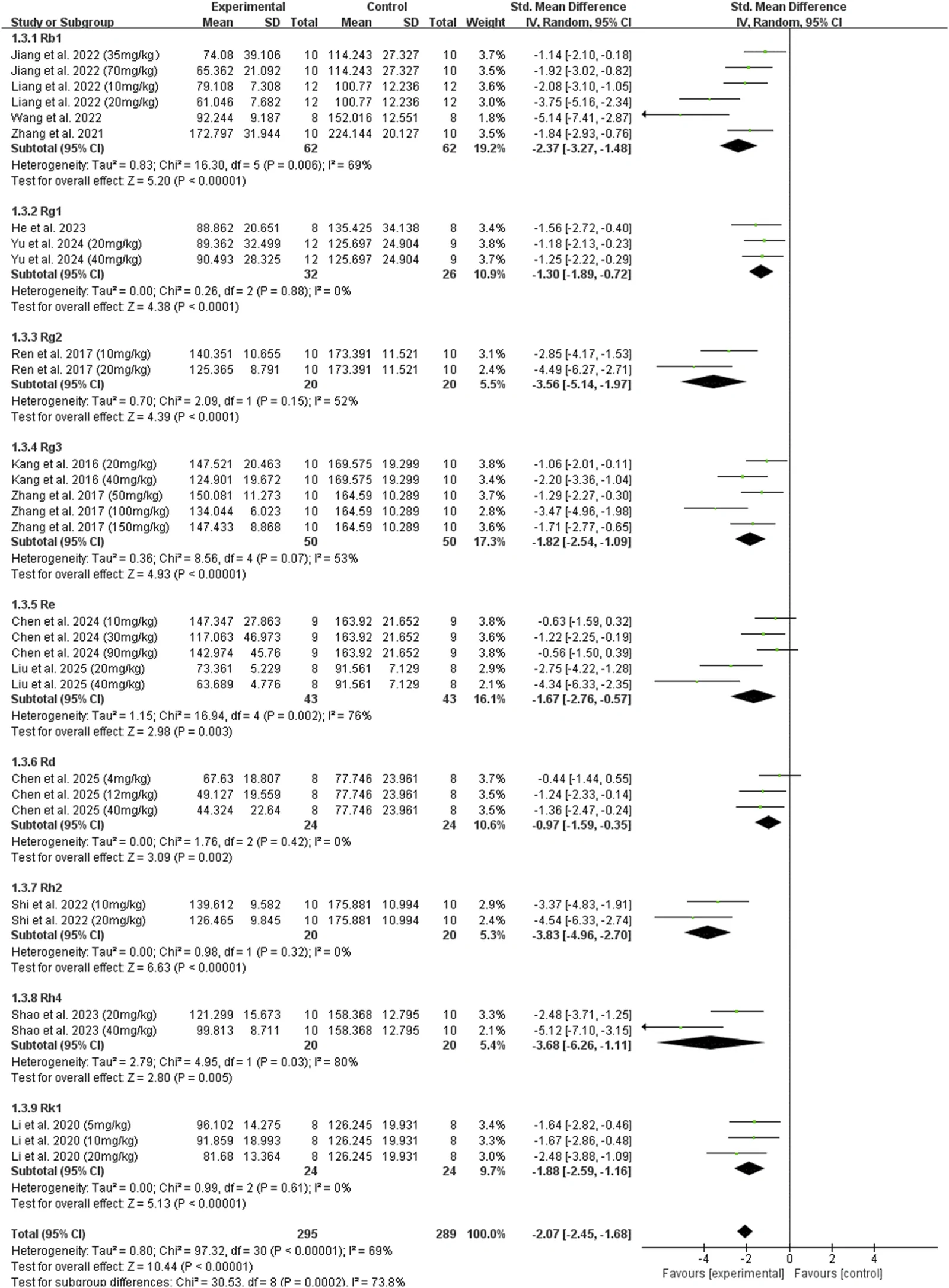
Forest plot for the effect of ginsenoside monomers and total ginsenosides on the number of rearing in the OFT. In this representation, an effect size with a 95% CI greater than 0—indicated by a horizontal line that lies entirely to the right of the vertical null line without intersecting it—signifies a significantly higher outcome rate in the treatment group compared to the model group. The meta-analysis demonstrated that some treatment groups significantly increased the number of crossings in model animals (SMD: 1.09, 95% CI: 0.54–1.64, Z = 3.86, *P* = 0.0001). Significant heterogeneity was observed across the included studies (I^2^ = 74%, *P* < 0.00001).

### Subgroup analyses of the dose-response relationship for GRb1

Because GRb1 showed comparatively large pooled effects in several behavioral domains, we performed a dose-stratified subgroup analysis to explore whether its apparent efficacy varied across dose levels. The results suggested different patterns across behavioral endpoints rather than a uniform dose-response relationship.

In the SPT, the high-dose subgroup showed a numerically greater effect than the low-dose subgroup, but the between-subgroup difference did not reach statistical significance (SMD = 4.83, 95% CI: 3.17–6.49; *p* = 0.07) (Figure 9A). By contrast, different patterns were observed in the FST and TST, in which lower immobility time indicates a stronger antidepressant-like effect. In both tests, the high-dose GRb1 subgroup showed numerically smaller effect sizes than the low-dose subgroup. Specifically, the magnitude of improvement appeared weaker in the high-dose subgroup in the FST (SMD = −2.92, 95% CI: −3.92 to −1.93; *p* = 0.02) and TST (SMD = −2.37, 95% CI: −3.27 to −1.48; *p* = 0.05) (Figures 9B,C). These findings suggest that the apparent dose-response profile of GRb1 may vary according to behavioral endpoint; however, given the limited number of studies within each subgroup and the heterogeneity of the underlying experiments, these results should be interpreted cautiously.

**FIGURE 9:**
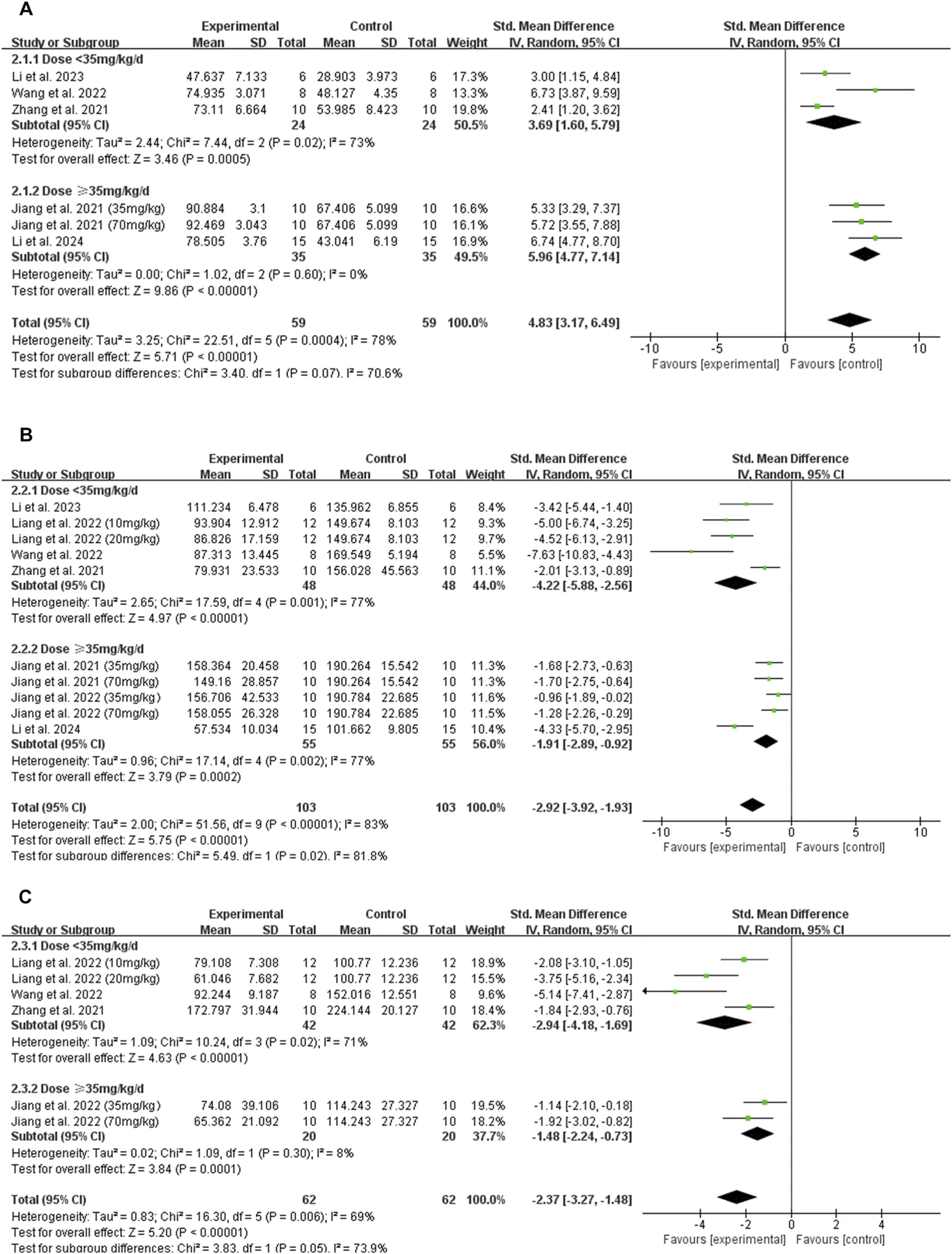
Subgroup analyses are performed for the effects of low-dose (<35 mg/kg/day) and high-dose (≥35 mg/kg/day) GRb1 in the SPT, TST, and FST. **(A)** Forest plot for the effect of GRb1 on the SPT. The meta-analysis demonstrated that both high- and low-dose treatment groups effectively increased sucrose solution consumption in model animals (SMD: 4.83, 95% CI: 3.17–6.49, Z = 5.71, *P* < 0.00001), with no statistically significant difference observed between the subgroups (*P* = 0.07). Significant heterogeneity was noted across the included studies (I^2^ = 78%, *P* = 0.0004). **(B)** Forest plot for the effect of GRb1 on the FST. The meta-analysis demonstrated that both high- and low-dose treatment groups significantly reduced the immobility time in model animals (SMD: −2.92, 95% CI: −3.92 to −1.93, Z = 5.75, *P* < 0.00001), with statistically significant difference observed between the subgroups (*P* = 0.02). Significant heterogeneity was noted across the included studies (I^2^ = 83%, *P* < 0.00001). **(C)** Forest plot for the effect of GRb1 on the TST. The meta-analysis demonstrated that both high- and low-dose treatment groups significantly reduced the immobility time in model animals (SMD: −2.37, 95% CI: −3.27 to −1.48, Z = 5.20, *P* < 0.00001), with statistically significant difference observed between the subgroups (*P* = 0.05). Significant heterogeneity was noted across the included studies (I^2^ = 69%, *P* = 0.006).

## Discussion

### Principal findings and incremental contribution

This systematic review and meta-analysis synthesized 25 animal studies evaluating 10 ginsenoside monomers and total ginsenosides. Across the available experiments, ginsenoside treatment improved anhedonia-like behavior, reduced immobility-related behavior, and altered selected measures of locomotor or exploratory activity. The clearest pooled findings were observed for the SPT, FST, and TST. GRb1 showed comparatively large pooled effects in the SPT and FST, GRh2 showed strong effects in behavioral-despair paradigms, and several other monomers showed notable effects in specific outcomes.

The principal contribution of this study is not the demonstration that ginsenosides have antidepressant-like activity, which has been reported previously (Kim and Cho, 2021; Ren et al., 2026), but the organization of that evidence by compound and behavioral domain. Interaction analyses provide a formal exploratory test of whether pooled effects differ across components and outcomes. Their practical value is to identify informative candidates and outcome domains for future direct comparisons. Because the underlying evidence consists of separate experiments rather than a connected network of head-to-head studies, these analyses refine the research agenda but do not establish a therapeutic ranking.

### Interpreting component-specific behavioral patterns

The apparent behavioral profiles should be interpreted as comparative patterns within the current literature. GRb1 was represented across several behavioral domains and showed large pooled effects in the SPT and FST, whereas GRh2 appeared particularly active in immobility-based tests. GRh4, GRg2, GRg1, and GRd also showed notable effects in selected analyses. These findings indicate that the evidence is not uniform across compounds or outcomes, but they do not demonstrate that one monomer is intrinsically more potent than another.

Between-compound differences may reflect genuine pharmacological variation, including differences in structure, metabolism, bioavailability, blood-brain barrier exposure, and molecular targets (Hu et al., 2023; Lipinski et al., 2001). They may also reflect differences in species, strains, depression models, dosing schedules, treatment duration, laboratories, and behavioral protocols (Kim and Cho, 2021). Moreover, the SPT, FST, TST, and OFT capture partially overlapping but non-equivalent behavioral constructs (Romano and Menzies, 2025). A large pooled effect in one test should therefore not be interpreted as broad superiority across depressive phenotypes. Claims linking individual ginsenosides to specific symptom clusters, reward circuits, or neural pathways remain premature unless these hypotheses are tested using direct mechanistic and behavioral comparisons.

### Robustness of effect estimates and dose-stratified findings

Several pooled SMDs were unusually large, including the overall SPT estimate and some component-specific and OFT estimates. Such values should not be read as direct measures of clinical magnitude. In small animal experiments, SMDs can be amplified by small within-group standard deviations, low sample sizes, selective reporting, methodological bias, and variation in test implementation. Inadequate reporting of randomization, allocation concealment, and blinding may further exaggerate apparent treatment effects. Funnel-plot asymmetry, when present, may reflect publication bias, but it may also arise from true heterogeneity, small-study effects, or the mathematical relation between SMDs and their standard errors (Friis Berntsen et al., 2021; Zwetsloot et al., 2017).

The precision of pooled estimates may also be affected when a single experiment contributes multiple doses, treatment arms, or behavioral outcomes. Analyzing each outcome separately reduces some dependence, but comparisons that share animals or control groups remain correlated unless that structure is explicitly modeled. The interaction analyses should therefore be viewed as exploratory, particularly when subgroup estimates are based on few studies (Brookes et al., 2001; Fingerhut et al., 2024). In addition, comparisons among ginsenoside components were predominantly indirect. Because the interventions represented distinct ginsenoside compounds, between-component heterogeneity is biologically plausible and may reflect genuine differences in pharmacological effects as well as differences in study design. Accordingly, heterogeneity across component groups should not be interpreted as evidence that a single study-level characteristic explains the observed variation, nor should indirect differences in pooled estimates be taken as evidence of comparative superiority.

The sparse and uneven distribution of studies across compound-model-outcome combinations precluded reliable meta-regression. Methodological guidance generally advises against meta-regression when fewer than 10 studies are available and recommends approximately 10 studies for each characteristic modelled; even this number may be insufficient when covariates are unevenly distributed across studies. In the present review, the number of independent studies within individual ginsenoside-outcome strata was below this threshold. Meta-regression was therefore not undertaken, and the absence of a formal moderator analysis should not be interpreted as evidence that important biological or methodological effect modifiers are absent. The GRb1 dose-stratified analysis should likewise be regarded as exploratory because the number of independent studies was limited.

The GRb1 dose-stratified analysis should likewise be regarded as hypothesis-generating. The division at 35 mg/kg is an analytical grouping rather than an established pharmacological threshold, and the available studies do not demonstrate a monotonic dose-response relationship. The observed subgroup pattern does not support a mechanistic conclusion that higher doses selectively improve anhedonia whereas lower doses preferentially affect behavioral despair. A defensible interpretation is limited to the observation that pooled effects varied across the available dose strata, with substantial uncertainty and potential confounding by model, route, duration, and study design.

### Mechanistic interpretation and strength of evidence

The mechanistic evidence was interpreted in light of the extent to which individual findings had been independently replicated. Mechanisms reported consistently across multiple independent studies were considered comparatively better supported, whereas those derived from a single study or a limited number of experimental observations were regarded as preliminary and should not be interpreted as established causal pathways.

Collectively, the included studies implicate several biological processes relevant to depression. Among these, the most recurrent findings involved the regulation of neuroplasticity-related signaling, particularly BDNF/TrkB-associated pathways, together with attenuation of neuroinflammatory and oxidative-stress responses (Chen et al., 2017; Correia et al., 2023; Liang et al., 2019). These mechanisms are biologically plausible and have been reported across more than one ginsenoside and experimental model, making them the most consistently observed mechanistic signals in the current evidence base. However, most studies assessed selected molecular markers following behavioral testing, and relatively few directly examined whether the implicated pathways were necessary for the observed behavioral effects through pharmacological blockade, genetic manipulation, or rescue experiments. Accordingly, the available evidence supports an association between these pathways and antidepressant-like effects but remains insufficient to establish a definitive causal role.

Evidence supporting NLRP3 inflammasome inhibition, SIRT1/Nrf2 signaling, HPA-axis normalization, synaptic remodeling, and gut-brain axis modulation is also promising, although it is less consistent across studies. Several of these pathways have been investigated in only a limited number of experiments or within a single compound-model context, which restricts the extent to which the findings can be generalized. Gut microbiota findings, in particular, are highly sensitive to differences in housing conditions, diet, sequencing methods, and analytical procedures (Gacesa et al., 2022; Hylander et al., 2023), while HPA-axis measurements may vary according to the timing of stress exposure and sample collection. These mechanisms are therefore more appropriately regarded as proposed pathways, or as mechanisms supported within specific experimental models, rather than as established class-wide effects. Overall, the current literature supports a convergent, multi-pathway framework for the antidepressant-related effects of ginsenosides, but it remains unclear which mechanisms are compound-specific, which have been independently replicated, and which are causally dominant.

### Translational implications and research priorities

The findings support continued investigation of ginsenosides as sources of antidepressant lead compounds, but the translational boundary is clear. All included evidence was derived from animal models, and antidepressant-like changes in rodent behavioral tests cannot be equated with symptom improvement in patients with major depressive disorder. The analysis provides no evidence of clinical efficacy, comparative effectiveness against established antidepressants, long-term safety, or an optimal human dose. Differences in absorption, metabolism, active metabolites, brain exposure, and species-specific pharmacology may substantially alter the effects observed in rodents (Gamo et al., 2017; Hendrie et al., 2013; Kinatukara et al., 2026).

GRb1 warrants further study because it has a relatively broad preclinical evidence base and showed large pooled effects in several outcomes, not because it has been proven to be the best ginsenoside. Priority experiments should directly compare GRb1, GRg1, GRh2, and other active monomers under the same model, dose-exposure framework, and behavioral protocol, with an established antidepressant as a benchmark. Studies should be prospectively powered, preregistered where possible, include both sexes, report randomization and blinded outcome assessment, and distinguish biological replicates from repeated measurements. Pharmacokinetic exposure, blood-brain barrier penetration, target engagement, active metabolites, safety margins, and reproducibility across laboratories should be established before clinical development is considered.

### Strengths and limitations

This review integrates evidence across multiple ginsenoside components, depression models, and behavioral domains, providing a broader comparative perspective than analyses focused on a single compound or outcome. However, several limitations should be considered. First, most comparisons were indirect and affected by substantial biological and methodological heterogeneity, while the limited number of independent studies in many compound–outcome combinations precluded adequately powered moderator analyses. Second, small sample sizes, incomplete reporting of randomization and blinding, possible selective reporting, and unusually large standardized mean differences may have inflated the pooled estimates. Third, multiple treatment arms, doses, and outcomes derived from the same experiments may have introduced residual statistical dependence. In particular, shared control groups and potentially correlated effect sizes were not formally adjusted for, which may have led to underestimation of uncertainty in some pooled estimates. Sparse dose-response data, limited pharmacokinetic information, and the absence of clinical studies further restrict dose-related and translational inferences.

Assessment of small-study effects and publication bias was also limited by the available evidence. Tests for funnel-plot asymmetry, including Egger’s test, have low power when fewer than 10 studies are available. In addition, when SMDs are used, effect estimates may be correlated with their standard errors, potentially producing spurious asymmetry (Sterne et al., 2011). Egger’s test was therefore not performed when the study-count criterion was not met, and funnel plots were interpreted descriptively rather than as definitive evidence of publication bias. This caution is particularly relevant to animal studies with small samples and multi-arm designs, where heterogeneity and residual dependence may also contribute to apparent small-study effects.

Geographical origin and cultivation environment may represent an additional source of heterogeneity in studies using ginseng-derived materials. Metabolomic analyses have shown regional variation in ginsenoside and other metabolite profiles of Panax ginseng. Such compositional differences may contribute to variation in pharmacological activity and experimental outcomes when source materials or standardization procedures differ across studies. This highlights the importance of standardized sourcing, authentication, and quality control in future preclinical research.

## Conclusion

Ginsenoside monomers and total ginsenosides were associated with improvements in several depression-related behavioral outcomes in animal models. The pooled evidence indicates beneficial effects on anhedonia-like behavior, immobility-related behavior, and selected measures of locomotor or exploratory activity. GRb1 showed comparatively large pooled effects in the SPT and FST, whereas GRh2, GRh4, GRg2, GRg1, and GRd showed notable effects in particular behavioral domains. These findings identify comparative patterns within a heterogeneous preclinical literature; they do not establish that any monomer is superior to other ginsenosides or to established antidepressants. GRb1 should therefore be considered a candidate for standardized head-to-head preclinical investigation rather than an established antidepressant therapy. Direct comparative experiments, rigorous control of bias, exposure-based dose assessment, pharmacokinetic and safety evaluation, and independent replication are required before the clinical relevance of GRb1 or other ginsenosides can be determined.

## Data Availability

The original contributions presented in the study are included in the article/supplementary material, further inquiries can be directed to the corresponding authors.
